# Metformin Impairs Spatial Memory and Visual Acuity in Old Male Mice

**DOI:** 10.14336/AD.2016.1010

**Published:** 2017-02-01

**Authors:** Nopporn Thangthaeng, Margaret Rutledge, Jessica M. Wong, Philip H. Vann, Michael J. Forster, Nathalie Sumien

**Affiliations:** Center for Neuroscience Discovery, Institute for Healthy Aging, University of North Texas Health Science Center at Fort Worth, Fort Worth, TX 76107 USA.; Center for Neuroscience Discovery, Institute for Healthy Aging, University of North Texas Health Science Center at Fort Worth, Fort Worth, TX 76107 USA.; Center for Neuroscience Discovery, Institute for Healthy Aging, University of North Texas Health Science Center at Fort Worth, Fort Worth, TX 76107 USA.; Center for Neuroscience Discovery, Institute for Healthy Aging, University of North Texas Health Science Center at Fort Worth, Fort Worth, TX 76107 USA.; Center for Neuroscience Discovery, Institute for Healthy Aging, University of North Texas Health Science Center at Fort Worth, Fort Worth, TX 76107 USA.; Center for Neuroscience Discovery, Institute for Healthy Aging, University of North Texas Health Science Center at Fort Worth, Fort Worth, TX 76107 USA.

**Keywords:** metformin, antidiabetic, aging, cognition, visual function, redox stress, antioxidant enzymes

## Abstract

Metformin is an oral anti-diabetic used as first-line therapy for type 2 diabetes. Because benefits of metformin extend beyond diabetes to other age-related pathology, and because its effect on gene expression profiles resembles that of caloric restriction, metformin has a potential as an anti-aging intervention and may soon be assessed as an intervention to extend healthspan. However, beneficial actions of metformin in the central nervous system have not been clearly established. The current study examined the effect of chronic oral metformin treatment on motor and cognitive function when initiated in young, middle-aged, or old male mice. C57BL/6 mice aged 4, 11, or 22 months were randomly assigned to either a metformin group (2 mg/ml in drinking water) or a control group. The mice were monitored weekly for body weight, as well as food and water intake and a battery of behavioral tests for motor, cognitive and visual function was initiated after the first month of treatment. Liver, hippocampus and cortex were collected at the end of the study to assess redox homeostasis. Overall, metformin supplementation in male mice failed to affect blood glucose, body weights and redox homeostasis at any age. It also had no beneficial effect on age-related declines in psychomotor, cognitive or sensory functions. However, metformin treatment had a deleterious effect on spatial memory and visual acuity, and reduced SOD activity in brain regions. These data confirm that metformin treatment may be associated with deleterious effect resulting from the action of metformin on the central nervous system.

Metformin (1,1-dimethylbiguanide) is currently the most widely prescribed drug for treatment of type 2 diabetes [1, 2]. The commonly accepted mechanism of the anti-hyperglycemic effect of metformin is suppression of hepatic glucose production via activation of 5′ adenosine monophosphate-activated protein kinase (AMPK), the primary energy sensor and regulator of energy homeostasis [3, 4]. Other mechanisms thought to contribute to therapeutic effects include the inhibition of mitochondrial respiratory chain complex I [5, 6] and mitochondrial glycerol 3-phosphate dehydrogenase [7], and decreased absorption of glucose from the intestines [8]. Furthermore, metformin may relieve hyperglycemia indirectly by improving insulin signaling [9] via gut microbiota changes [10] and activation of NF-κB [11].

Metformin is an effective treatment for conditions other than type 2 diabetes, including polycystic ovarian syndrome and cancers [12-16], and it is associated with lower morbidity and mortality from cardiovascular diseases [17, 18]. The broad benefits of metformin treatment have contributed to the suggestion that metformin’s effects are similar to those of long term caloric restriction (CR), an intervention that not only improves insulin sensitivity and reduces blood glucose, but is well known to delay age-related pathology and robustly extend life span of mammals. Indeed, the gene array profile after metformin treatment resembles that of CR [19, 20] and both interventions may effect an activation of the SKN-1/Nrf2 transcription factor [21] involved in redox homeostasis regulation [22] and AMPK activation [21, 23]. Furthermore, metformin supplementation is reported to extend life span in both *C. elegans* [21] and mice [24-26]. Based on its broad therapeutic actions and the strong parallel with CR, a clinical trial of metformin as an “anti-aging” intervention has been described (Metformin in Longevity Study, NCT02432287) [27].

The pharmacokinetics of metformin have been well described in humans [28] and it has been shown to cross the blood-brain barrier upon acute and chronic administration [29]. Studies of the effects of metformin on central nervous system function and pathology have yielded conflicting outcomes [30], with some results suggesting enhanced neurogenesis, improved spatial learning [31] and reduced risk of cognitive declines [32]; other studies have indicated negative consequences such as an increase in risk for Alzheimer’s disease (AD), cognitive dysfunction [33, 34], impaired neuronal survival [35] and exacerbation of cognitive dysfunction in a mouse model of AD [36]. Furthermore, beneficial effects of metformin have been associated with its antioxidative capacity in type 2 diabetic patients [37, 38]. Metformin effects have implicated the activation of Nrf2, which acts as an antioxidant regulator via its nuclear translocation and activation of antioxidant response elements thereby regulating redox homeostasis enzymes [22]. Studies in mice fed high fat diets related decreased Nrf2 mRNA when supplemented with metformin for 4 months [39]. Furthermore, other studies have reported that short-term treatment with metformin restored erythrocyte age-dysregulated redox status [40]. Taken together, these studies paint an unclear picture of the effects of metformin on brain function and redox homeostasis.

The current studies addressed ambiguity in the previous preclinical literature on brain function, through application of a comprehensive approach that involved testing metformin in the context of different domains of cognitive, sensory and psychomotor function in different age groups of mice. An oral metformin treatment regimen was used that was calculated to mimic a clinically relevant dose of metformin in young-mature, middle-aged and old mice. These age groups were selected to model different life periods in humans when treatment would be initiated for relief of diabetic symptoms, diabetes prevention, or for delay of biological aging. The mice were given metformin for one month prior to the start of the functional assessments, and this treatment continued throughout the testing for a total of 3 months. Tissues were collected at the end of these studies and used for measurement of enzyme activities relevant to maintenance of redox homeostasis.

## MATERIALS AND METHODS

### Animals and treatments

Procedures pertaining to animal handling and maintenance adhered to the NIH guidelines and were approved by the UNT Health Science Center Institutional Animal Care and Use Committee. Three age groups of adult male C57BL/6J mice (4 mo, *n*=32; 11 mo, *n*=32; 22 mo, *n*=36) were obtained from the National Institute on Aging colony (supplied by Charles River). Mice were group housed (3-4/cage) in clear polycarbonate cages at 23 ± 1°C under a 12-h light/dark cycle starting at 0600. Half of the mice from each age group were assigned to a control group, and the other half to a metformin group in which metformin (Sigma Aldrich, St. Louis, MO) was added to the drinking water (2 mg/ml). Taking into account the body weights of the mice, it was estimated that mice received ~219-297 mg/kg/day (Table 1), amounts which are comparable to human doses of 1500-2000 mg/day [41]. The mice were maintained on their respective treatments for the duration of the study (3 months) and body weights, and food and fluid intakes were measured daily. Blood glucose levels were measured at 4 pm before metformin treatment began, and after 3 months of treatment using a glucose-monitoring system by FreeStyle Lite (Abbott Diabetes Care Inc., Alamenda, CA).

### Functional measures

After 1 month of treatment, a behavioral test battery was administered that assessed spontaneous locomotor activity, musculoskeletal reflexes (walking initiation, alley turning, negative geotaxis), strength and balance (wire suspension, bridge walking), cognitive performance (Morris water maze, discriminated avoidance reversal), and visual capacity (optomotor task). The performance of mice on these tests has been shown to decline with age [42-44].

**Table 1:** Physiological assessments for age and treatment groups.

	Young		Middle-age		Old	

	Control	Metformin	Control	Metformin	Control	Metformin
**Body weight at the start of the study** (g)	27.85 ± 0.58	26.98 ± 0.35	33.69 ± 0.69^*^	32.78 ± 0.67^*^	34.21 ± 0.50^*^	33.89 ± 0.63^*^
**Body weight at the end of the study** (g)	30.36 ± 0.46	29.687 ± 0.47	37.86 ± 1.10^*^	35.05 ± 0.88^*#^	34.46 ± 0.68^*^	33.57 ± 0.75^*^
**Food intake across study duration** (g/day)	4.13 ± 0.22	3.57 ± 0.08^#^	3.98 ± 0.08	3.86 ± 0.09	4.55 ± 0.18^*^	4.15 ± 0.10^*#^
**Water intake across study duration** (ml/day)	4.73 ± 0.42	4.17 ± 0.09	3.93 ± 0.07	3.72 ± 0.08	4.62 ± 0.27	4.44 ± 0.28
**Percent change in blood glucose from the start to the end of the study** (mg/dl)	-3.99 ± 7.47	-0.65 ± 6.80	-11.17 ± 4.59	2.95 ± 6.69	-9.92 ± 5.26	-4.85 ± 5.71
**Calculated daily metformin dose** (mg/kg)	0	298.7	0	219.0	0	259.6

Each value represents the mean ± SE of young (5 mo), middle-aged (13 mo), or old (24 mo) mice.

* significantly different from treatment-matched young, *p*<0.05

# significantly different from age-matched controls, *p*<0.05

#### Spontaneous activity

During a 16-min test period, a mouse’s movements in the horizontal plane as well as the vertical plane were detected by the photocells and processed by software, yielding variables that described horizontal, vertical, stereotypic, and spatial components of spontaneous activity.

#### Musculoskeletal reflexes

Over four consecutive daily sessions, the mice were administered three simple reflex tests. The first test consisted of placing the mouse on a flat smooth surface and recording the latency to move one body length (walk initiation). The second test measured the latency to reverse direction when the mouse was placed in a dead-end alley (alley turning). For the third test, the mouse was placed facing downward on a flat surface that was tilted 45°, and the latency to turn 90° in either direction was measured (negative geotaxis). Latencies were recorded and averaged over the four sessions.

#### Wire suspension

Mice were administered the wire suspension test for four consecutive days (2 trials/day). For each trial (lasting a maximum of 60s), the mouse was allowed to grip a horizontal wire with the front paws and the latency to tread (grasp the wire with the mouse’s hind legs) and the latency to fall were recorded and averaged over the four sessions.

#### Bridge walking

Each mouse was tested for the latency to fall or reach a safe platform after being placed in the middle of one of four acrylic bridges that differed in diameter (small or large) and shape (round or square) so as to provide four incremental levels of difficulty. Each bridge was presented three times, and the measure of performance was the average latency to fall (up to a maximum of 60 s) across all bridges.

#### Spatial learning and memory

Spatial learning and memory were measured using a swim maze test as described previously [43]. The performance of the mice was measured in a series of 9 training sessions, during which the platform location remained in a fixed site. Spatial bias for the platform was assessed on the last trial of sessions 2,4,5,6, and 9 and 7 days after session 9 via a probe trial during which the platform was removed. The amount of time spent in the annulus 40 cm around the platform location was used to measure spatial bias.

#### Discriminated avoidance test

A T-maze constructed of acrylic (black for the sides and clear for the top) was utilized for the discriminated avoidance task [43, 44]. The ability of the mice to learn the avoidance problem was considered inversely proportional to the number of trials required to reach criterion in each of the sessions. The mice were trained until they reached the criterion of correct avoidance (defined as running directly to the correct arm within 5 s) on four of the last five training trials, with the last two being correct.

#### Optomotor task

The testing apparatus was a chamber (39 x 39 x 32.5 cm) with mirrored floors and ceilings. Attached to each of the four walls was a 20-in computer monitor facing inwards. In the center of the chamber was a platform (7-cm diameter) that was elevated approximately 15 cm from the floor. When a mouse was placed on the platform, a video camera positioned in the ceiling of the apparatus enabled the mouse’s behaviors to be clearly visible during testing. A computer program was used to project visual stimuli (vertical gratings) onto the monitors (OptoMotry, CerebralMechanics, Lethbridge, Alberta, Canada) [45]. The gratings were then rotated at 12 degrees/second, producing the appearance of a virtual rotating cylinder. The moving gratings elicited a tracking behavior and the visual acuity threshold was determined for each eye by projecting a grating of low spatial frequency (0.042 cycles/degree) onto the walls, rotating in a clockwise direction (testing the left eye) or in the counterclockwise direction (testing the right eye) and set at the highest spatial frequency to which the animal responded. A staircase method of determining acuity threshold was implemented, such that a series of gratings of increasingly higher spatial frequencies was presented (rotating in one, then the alternative direction) as long as the mouse indicated that it could detect the grating movements. The mean visual acuity was calculated as the average acuity of both eyes.

### Tissue preparation

The mice were euthanized via cervical dislocation followed by decapitation and each brain was dissected and cortex and hippocampus were collected, and liver was also harvested. These samples were homogenized in antioxidant buffer (10 mM sodium phosphate, 0.9% sodium chloride, 200 μM DTPA and 1 mM BHT) and protease inhibitors cocktail (Roche Diagnostics, Indianapolis, IN), and centrifuged at 21,730 x g for 30 min at 4° C. The supernatants were aliquoted for subsequent enzyme activity assays.

### Enzyme activity assays

The activity of superoxide dismutase and enzymes involved in the homeostasis of glutathione were measured in liver, cortex and hippocampus to determine whether age and metformin treatment had any effect. Superoxide dismutase (SOD). The activity was detected by observing the oxidation of NADPH at 340 nm. In the presence of SOD, the nucleotide oxidation is inhibited, thus leaving the absorbance at 340 nm unchanged. Activity of SOD in the sample was determined according to the method described by Paoletti and Mocali [46]. One unit of SOD activity is defined as the amount of SOD required to inhibit the rate of NADPH oxidation by 50%. Glutathione reductase (GR). The activity was determined by measuring the increase in absorbance at 412 nm, which occurred as the result of 5, 5′-dithiobis (2-nitrobenzoic acid) (DTNB) being reduced by glutathione (GSH). In brief, the reaction was initiated by adding 80 ug of homogenate to reaction buffer (90 mM KPO_4_, 0.45 mM EDTA, and 0.75 mM DTNB) containing either 98.4 µM NADPH (sample) or without NADPH (blank). Change in absorbance was monitored at 412 nm for 5 min at room temperature. GR activity in the sample was calculated by subtracting blank from sample and expressed as U/mg protein [47]. Glutathione peroxidase (GPx). The activity was indirectly measured via a coupled reaction with glutathione reductase and NADPH to regenerate GSH from GSSG as described in [48]. Upon addition of 250 ug of homogenate, the decrease in the absorbance at 340 nm over 5 min was observed and used to calculate GPx activity (expressed as U/mg protein). Glutaredoxin (Grdx). Using the standard β-hydroxyethyl disulfide, the activity of GSH-dependent Grdx activity was assessed, as described by Lu et al. [49]. Briefly, 75-80 µg of homogenate was used to initiate the reaction. The activity of Grdx was indirectly measured from the decrease in the absorbance at 340 nm for 5 min, and expressed as 1 nmol NADPH utilized/mg protein. Thioredoxin reductase (TrxR). The activity was determined by utilizing the reduction of *E. coli* thioredoxin by NADPH. The reduced form of thioredoxin is reoxidized by disulfides from insulin which produce sulfhydryl groups in insulin. The reaction was initiated by the addition of 50-70 ug of homogenate to reaction buffer containing 85 mM HEPES, 34 mM EDTA, 0.68 mg/ml NADPH, 2.14 mg/ml bovine insulin with 5 µM *E. coli* thioredoxin (sample) or without *E. coli* thioredoxin (blank). Each sample was done in duplicate. The reaction was allowed to continue for 1 hour at room temperature, after which 500 µl of stopping solution (6 M guanidine hydrochloride and 0.4 mg/ml DTNB) was added to each tube. After a brief vortex, the solution was measured at 412 nm. The activity of thioredoxin reductase was obtained by subtracting blank from sample and expressed as U/mg protein [50, 51]. Glutathione S-transterase (GST). The activity of GST in the sample was measured according to Mannervik [52] and Boyland and Chasseaud [53]. In brief, 30 µg of the homogenate was added to phosphate-buffered saline (pH 6.5) containing 1 mM 1-chloro-2, 4-dinitrobenzene and 1 mM glutathione (GSH). Each sample was done in duplicate. The increase in the absorbance at 340 nm was monitored for 5 min. GST activity is expressed as U/mg protein. γ-Glutamate-cysteine ligase (GCL). GCL activity was determined by following a method described in Kim et al. [54]. Briefly, the reaction was initiated by adding 20 ug of the homogenate to 1.0 ml reaction mixture (100 mM Tris-HCl buffer (pH 8.2), 150 mM KCl, 5 mM ATP, 2 mM phosphopyruvate, 10 mM L-glutamate, 10 mM L-α-aminobutyrate, 20 mM MgCl_2_, 2 mM EDTA, 0.2 mM NADH, 1 U pyruvate kinase, 1 U lactate dehydrogenase). The rate of decrease in absorbance at 340 nm was monitored for 5 min at room temperature. GCL activity is expressed as µmoles of NADH oxidized/min/mg protein.

### Statistical analyses

The effects of Age and Treatment were assessed using two-way analyses of variance (ANOVA) for most measures. Measures of body weight and water maze performance were considered in three-way ANOVAs (with Time point or Session as repeated measures). Planned individual comparisons between metformin-treated and control groups at each age were performed using single degree of freedom F tests using the error term from the overall analysis. The alpha level was set at < <05 for all analyses.

**Table 2:** Effects of age and metformin treatment on simple reflexes and spontaneous locomotor activity

	Young		Middle-age		Old	

	Control	Metformin	Control	Metformin	Control	Metformin
**Latency to initiate walking** (s)	2.13 ± 0.43	2.38 ± 0.31	3.79 ± 0.47^*^	3.48 ± 0.58	3.36 ± 0.39	4.75 ± 0.56^*#^
**Latency to turn in a dead-end alley** (s)	8.82 ± 0.60	11.01 ± 1.66	14.03 ± 2.24	10.75 ± 1.34	16.56 ± 1.96^*^	18.18 ± 2.57^*^
**Latency to tread (wire suspension)** (s)	20.31 ± 3.55	21.75 ± 4.72	46.47 ± 4.30^*^	44.77 ± 4.35^*^	52.33 ± 3.04^*^	49.61 ± 2.94^*^
**Latency to negative geotaxis** (s)	2.39 ± 0.73	2.08 ± 0.29	2.34 ± 0.63	1.74 ± 0.17	2.06 ± 0.48	2.26 ± 0.25
**Distance traveled** (cm)	571.9 ± 36.8	585.9 ± 30.5	526.8 ± 25.9	548.9 ± 26.2	561.6 ± 34.1	624.8 ± 36.3

Each value represents the mean ± SE of 15-18 mice.

* Significantly different from treatment-matched young, *p*<0.05

# Significantly different from age-matched control, *p*<0.05

## RESULTS

### General assessment: body weight, food and water intake, blood glucose

Middle-aged and old mice weighed more than young mice overall and young and middle-aged mice, but not old mice, gained weight across the 3 months of the study. (Table 1). The middle-aged metformin-treated groups gained less weight than age-matched controls across the study, whereas there was no effect of treatment in the young or old groups. These observations were supported by a significant interaction between Time, Age and Treatment (*p*= 0.027). It should be noted that 1 of 18 old controls and 3 of 18 old metformin-treated mice died during the course of the experiment. There was no mortality in the young or middle-aged groups.

Food intake was 9-14% higher in old mice than young ones, and 9-16% lower in metformin-treated mice compared to controls in the young and old group (Table 1). A two-way ANOVA revealed significant main effects of Age and Treatment (all *p*s<0.006) but no significant interaction of these factors (*p*=0.382). Water intake tended to be lower in the middle-aged mice compared to young and old mice, and was not affected by metformin treatment. A two-way ANOVA yielded a significant main effect of Age (*p*=0.029), but no effect of Treatment or an interaction of Treatment and Age (all *p*s> 0.156).

**Figure 1.**
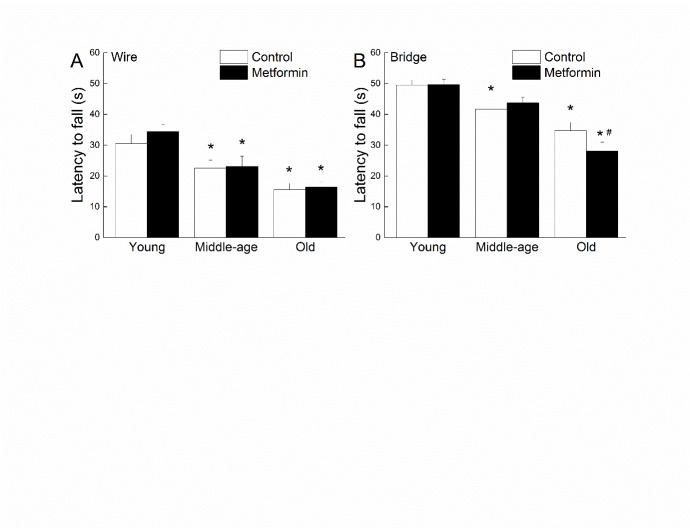
**Effects of age and metformin on strength and balance**. Effects of age and metformin treatment on wire suspension (**A**) and bridge walking (**B**) performance as measured by latency to fall in seconds. Each value represents the mean ± SE of groups composed of 16-18 mice. * denotes *p*<0.05 from treatment-matched young; # denotes *p*<0.05 from age-matched control.

**Figure 2.**
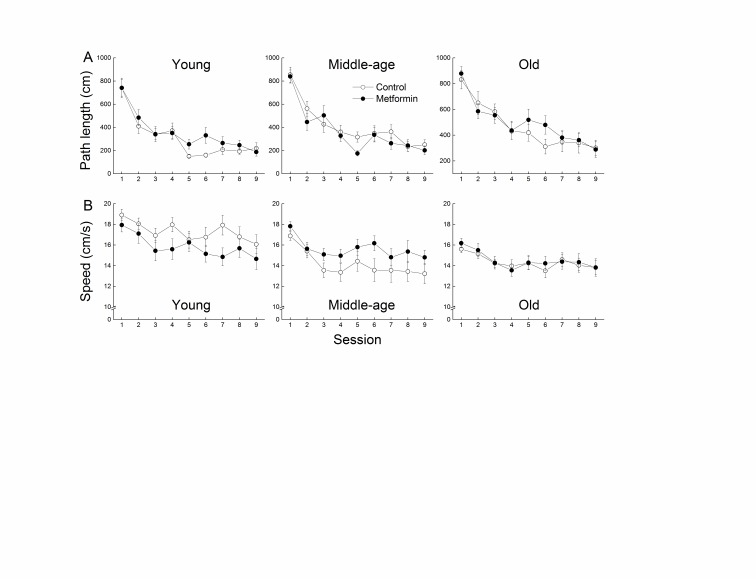
**Effects of age and metformin on spatial learning**. Effects of age and metformin treatment on Morris water maze performance as measured by path length (cm ± SE; **A**) taken to reach the hidden platform, and by speed (cm/s ± SE; **B**) during 9 sessions of acquisition. Each value represents the mean ± SE of groups composed of 16-18 mice.

Because metformin has been reported to effectively lower blood glucose in disease animal models [55, 56], blood glucose levels were measured prior to the beginning of metformin treatment and at the end of the treatment. The percentage of blood glucose change from the beginning to the end of the study was calculated in order to correct individual differences in baseline (Table 1). There was no effect of age or treatment on blood glucose level changes, as indicated by a lack of significant main effects and the interaction of Age and Treatment (all *p*s>0.139).

### Simple reflexes and motor functions

The reflex latency tests for walking initiation, alley turning and treading each revealed age-related slowing in middle-aged or old control groups (Table 2). Metformin treatment increased the latency to initiate walking in the old mice, but did not have any other notable effect on the age-sensitive reflexive measures. Two-way ANOVAs for each of these measures yielded a significant main effect of Age (all *p*s <0.001), but failed to detect a main effect of Treatment or an interaction between Age and Treatment (all *p*s >0.177). Negative geotaxis and spontaneous locomotion (distance traveled) were not affected by age or treatment (all *p*s> 0.211 in two-way ANOVAs).

The performance of mice during the wire suspension and bridge walking tests is depicted in Figure 1 as a function of age and treatment. The mean latency to fall showed a progressive, age-related, decrease in control groups for both motor tests and was 37 to 50% shorter than young control by old age. The old metformin-treated mice had a shorter latency to fall from the bridge when compared with their age-matched controls (Fig. 1 B), but no other effect of treatment was evident for the wire or bridge test. Two-way ANOVAs for the wire and bridge latency data yielded only significant main effects of Age (*ps*>0.001).

### Cognitive functions

The efficiency of the mice in learning the spatial swim maze task was assessed by the length of the path taken to reach the hidden platform (Fig. 2A) independently of the speed of swimming (Fig. 2B). While all groups learned to locate the platform more efficiently as a function of sessions, the path length taken by the mice was progressively longer as a function of age. A decrease in the path-independent swim speed was also evident as a function of age. Metformin treatment had no overall effect on path length or swim speed in any of the age groups. Three-way ANOVAs on path length and swim speed data yielded significant main effects of Session and Age (*p*s <0.001) but no effects of Treatment or an interaction between Age and Treatment (all *p*s>0.051).

**Figure 3.**
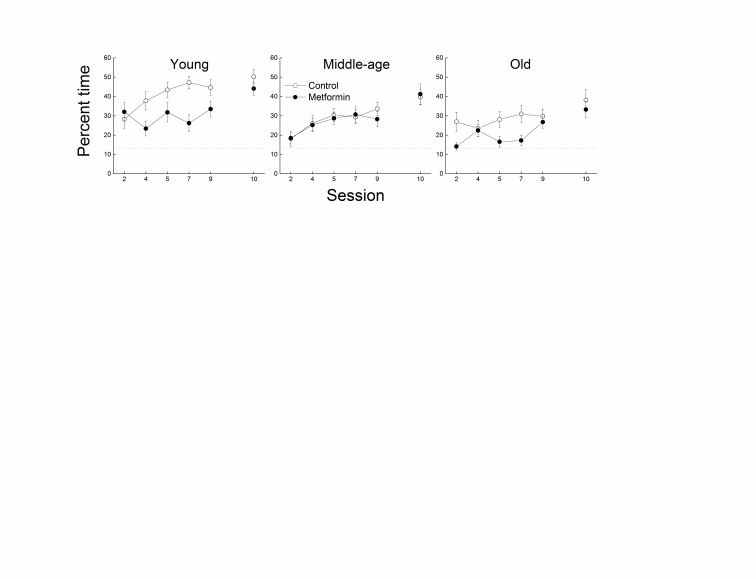
**Effects of age and metformin on spatial memory**. Effects of age and metformin treatment on spatial bias during the Morris water maze task, as measured by the percent time spent in an annulus 40-cm around the platform location. This probe trial during which the platform is not accessible is done on sessions 2,4,5,7 and 9 and on session 10 (7-day delay). Each value represents the mean ± SE of groups composed of 16-18 mice. The dotted line represents the % time spent in annulus 40cm due to chance.

Spatial memory accuracy was measured via probe trials conducted as the last trial of sessions 2, 4, 5, 7, and 9 (Fig. 3). Young, middle-aged and old control mice developed a strong bias for the platform location across sessions, as indicated by an increase in the percent time spent in the annulus 40 above chance level. However, bias for the platform location was markedly greater in the young group when compared with the middle-aged and old mice. Metformin-treated mice in both the young and old groups spent less time in the annulus 40 than their age-matched controls, though no effect of metformin was evident in the middle-aged group. A three-way ANOVA supported these observations, indicating significant effects of Session, Age, Treatment (all *p*s<0.002), and their three-way interaction (*p*=0.014). When mice were given a retention probe trial (Session 10) seven days following the last acquisition session, the effect of age on probe performance was still evident, but there was no effect of metformin treatment in any of the age groups. A separate analysis of the retention trial data revealed only a significant effect of Age (*p*=0.037).

**Figure 4.**
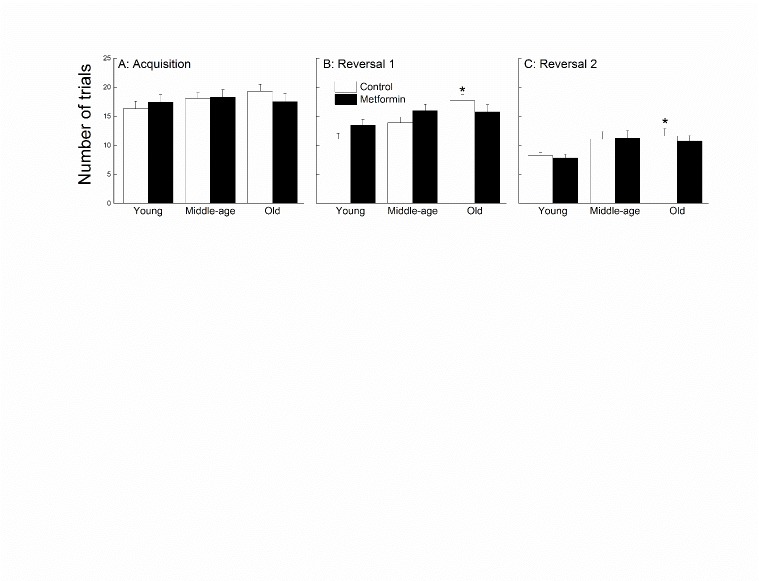
**Effects of age and metformin on learning and cognitive flexibility**. Effects of age and metformin treatment on discriminated avoidance task as measured by the number of trials taken to reach a criterion of 4 out 5 correct avoidances, with the last two being correct (A: acquisition; B and C: reversals). Each value represents the mean ± SE of groups composed of 15-17 mice. * denotes p<0.05 from treatment-matched young.

Discriminated avoidance learning was considered for effects of age and metformin treatment during one acquisition session (Fig. 4A) and two reversal sessions (Fig. 4B and C). During the acquisition session, there were no differences among the age or treatment groups, supported by a lack of effects or an interaction following a two-way ANOVA (all *p*s> 0.402). During the reversal sessions, the young mice took fewer trials to reach criterion compared to the middle-aged and old mice, and the metformin-treated mice performed similarly to the controls regardless of age. Analyses of these data yielded a significant effect of Age for both sessions (all *p*s<0.003), and a lack of Treatment effect or interaction (all *p*s>0.09).

**Figure 5.**
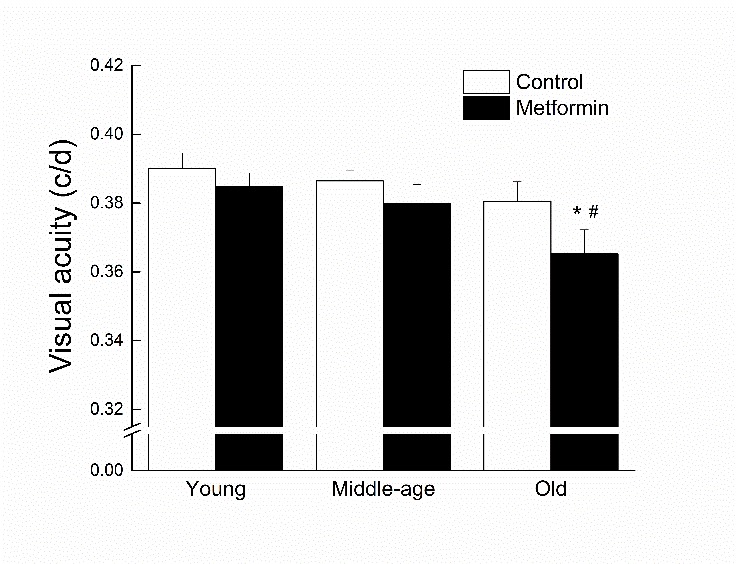
**Effects of age and metformin treatment on visual acuity, measured as the highest spatial frequency (in c/d) of visual stimuli to which the mouse responded**. Each value represents the mean ± SE of groups composed of 15-17 mice. * denotes p<0.05 from treatment-matched young; # denotes p<0.05 from age-matched control.

### Visual acuity

Visual acuity was measured as the highest spatial frequency of visual stimuli at which the optomotor reflex could be detected (Fig. 5). Old metformin-treated mice had lower visual acuity that their control counterparts, and a similar trend was present in the other age groups. There was also an overall decline in acuity as a function of age. Both trends contributed to main effects of Age and Treatment (all *p*s<0.034) when acuity data were considered in a two-way ANOVA.

### Activity of superoxide dismutase (SOD) in the liver, cerebral cortex and hippocampus

**Figure 6.**
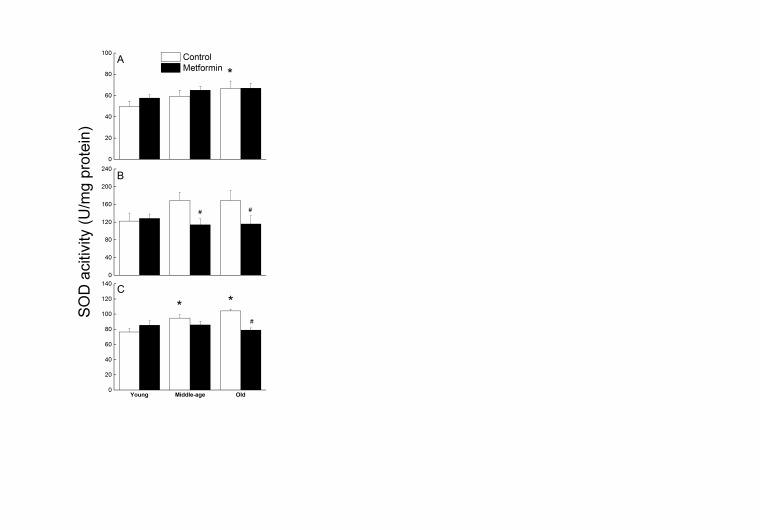
**Effects of age and metformin treatment on SOD activity (U/mg protein) in liver (A), cerebral cortex (B), and hippocampus (C)**. Each value represents the mean ± SE of 6-8 mice. * denotes p<0.05 from treatment-matched young; # denotes p<0.05 from age-matched control.

In the liver, SOD activity was increased in the old control mice compared to the young ones (Fig. 6A). There was no effect of metformin treatment on SOD activity at any age. These observations were supported by a significant main effect of Age (*p*=0.034) and a lack of main effect of Treatment or an interaction between Age and Treatment following a two-way ANOVA (all *p*s>0.255).

In the cerebral cortex and hippocampus, SOD activity was higher in the middle-aged and old mice compared to the young (Figure 6B and C, respectively). In the cortex, SOD activity was markedly lower in the metformin-treated mice than age-matched controls in the middle-age and old groups. In the hippocampus, this effect was present only in the old groups. Two-way ANOVAs revealed significant effects of Treatment for both regions (all *p*s<0.019), but no significant main effect of Age was detected (all *p*s>0.051). For SOD activity in the hippocampus, a significant Age x Treatment interaction was found (*p*=0.004).

### Activity of redox-homeostasis enzymes in the liver

The effects of age and metformin on the activity of redox-homeostasis enzymes in the liver is summarized in Table 3. Generally, there were no evident differences in the activity of GR, GPx, Grdx, GST, and GCL between any age group or between treatment groups (all *p*s > 0.112).

Young metformin-treated mice had a TrxR activity nearly double that of their controls, which was supported by a significant interaction between Age and Treatment and a main effect of age (*p*=0.009).

**Table 3:** Redox-homeostasis enzyme activity.

	Liver					
	Young		Middle-age		Old	
	Control	Metformin	Control	Metformin	Control	Metformin
**GR**	99.1 ± 26.1	145.1 ± 24.0	74.5 ± 21.2	74.5 ±17.5	125.9 ± 34.8	115.4 ± 31.7
**GPx**	399.8 ± 25.2	409.8 ± 14.4	407.2 ± 17.3	428.5 ±15.9	376.6 ± 18.1	413.3 ± 22.8
**Grdx**	5.2 ± 0.1	5.5 ± 0.2	5.4 ± 0.3	6.1 ± 0.2	6.4 ± 0.6	5.8 ± 0.5
**TrxR**	3.9 ± 0.54	6.7 ± 0.31^#^	3.7 ± 0.55	4.0 ± 0.47^*^	5.5 ± 0.66	4.8 ± 0.76^*^
**GST**	388.6 ± 19.6	433.9 ± 21.1	419.4 ± 16.4	399.8 ±10.5	453.1 ± 34.2	398.1 ± 39.1
**GCL**	59.9 ± 3.8	68.2 ± 2.5	68.3 ± 3.6	68.4 ± 4.7	68.1 ± 4.6	62.9 ± 3.3

Each value represents the mean ± SE of 4-8 mice.

**Abbreviations**: GR, Glutathione reductase activity (U/mg protein); GPx, Glutathione peroxidase activity (U/mg protein); Grdx, Glutaredoxin activity (1nmol NADPH utilized/min/mg protein); TrxR, Thioredoxin reductase activity (U/mg protein); GST, Glutathione S-Transferase activity (U/mg protein); GCL, Glutamate Cysteine Ligase activity (1 nmol NADH utilized/min/mg protein)

* Significantly different from treatment-matched young, *p*<0.05

# Significantly different from age-matched control, *p*<0.05

**Table 4:** Redox-homeostasis enzymes activity detailing group means and standard errors for enzymatic activity in the cerebral cortex and hippocampus

	Cerebral cortex						Hippocampus					

	Young		Middle-age		Old		Young		Middle-age		Old	

	Control	Metformin	Control	Metformin	Control	Metformin	Control	Metformin	Control	Metformin	Control	Metformin
**GR**	174.1 ± 9.5	174.2 ± 8.7	172.9 ± 5.9	187.8 ± 8.9	176.2 ± 5.6	193.2 ± 9.6	212.3 ± 13.3	203.0 ± 9.3	236.7 ± 20.8	225.8 ± 15.5	226.1 ± 32.1	203.7 ± 8.8
**GPx**	16.7 ± 1.1	19.0 ± 0.5^#^	19.2 ± 0.7^*^	19.1 ± 0.6	18.2 ± 1.1	18.3 ± 0.4	30.2 ± 3.2	30.7 ±2.3	30.6 ± 1.1	30.2 ± 2.2	34.5 ± 3.3	35.9 ± 2.5
**Grdx**	29.5 ± 0.9	29.2 ± 0.4	29.0 ± 0.5	29.2 ± 0.6	29.5 ± 0.7	29.4 ± 0.7	31.8 ± 1.2	34.0 ± 1.7	32.7 ± 0.8	34.9 ± 1.4	33.8 ± 2.1	34.4 ± 1.1
**TrxR**	11.8 ± 0.8	12.1 ± 0.7	11.2 ± 0.5	12.5 ± 0.4	12.4 ± 1.0	12.2 ± 0.5	14.5 ± 1.26	15.0 ± 2.60	16.0 ± 1.98	15.4 ± 1.50	18.4 ± 3.63	13.7 ± 1.30
**GST**	40.3 ± 2.6	48.9 ± 3.1^#^	53.7 ± 2.4^*^	55.2 ± 2.5	56.7 ± 4.3^*^	50.3 ± 3.1	177.9 ± 11.3	166.2 ± 11.4	197.5 ± 13.8	201.5 ±7.4	225.3 ± 25.4^*^	186.2 ± 9.5
**GCL**	71.5 ± 3.7	73.2 ± 3.5	79.5 ± 3.1	79.6 ± 3.8	81.4 ± 3.6	82.5 ± 3.8	N/A	N/A	N/A	N/A	N/A	N/A

Each value represents the mean ± SE of 4-8 mice.

**Abbreviations**: GR, Glutathione reductase activity (U/mg protein); GPx, Glutathione peroxidase activity (U/mg protein); Grdx, Glutaredoxin activity (1nmol NADPH utilized/min/mg protein); TrxR, Thioredoxin reductase activity (U/mg protein); GST, Glutathione S-Transferase activity (U/mg protein); GCL, Glutamate Cysteine Ligase activity (1nmol NADH utilized/min/mg protein)

* denotes significantly different from treatment-matched young, *p*<0.05

# denotes significantly different from age-matched control, *p*<0.05

### Activity of redox-homeostasis enzymes in the cerebral cortex and the hippocampus

Table 4 summarizes the activity of redox-homeostasis enzymes examined in the cerebral cortex and the hippocampus. Overall, the activity of GR, Grdx, and TrxR were unaffected by age or treatment in the cerebral cortex and hippocampus (all *p*s >0.128). GPx activity in the cortex did not appear to be affected by either age or treatment, although activity level of the young controls was less than the other groups. A one-way ANOVA did not yield any significant main effect or interaction (all *p*s>0.115). The activity of GST in the hippocampus was approximately 3 times higher when compared to the cerebral cortex. GST activity in both the cerebral cortex and the hippocampus generally increased with age. In the cortex, young metformin-treated mice had higher GST activity than young controls. One-way ANOVAs resulted in significant main effects of Age for both regions (all *p*s<0.038), but no effect of Treatment (all *p*s>0.163) or an Age x Treatment interaction (all *p*s <0.058). In the cerebral cortex, the activity of GCL increased with age regardless of treatment (*p* = 0.031) and was unaffected by metformin treatment (all *p*s>0.735). GCL activity was not determined in the hippocampus due to insufficient sample.

## DISCUSSION

The major findings of the study were that metformin supplementation in male mice: (i) failed to affect blood glucose levels, body weight or redox homeostasis at any age and did not yield beneficial effects against age-impaired psychomotor, cognitive or sensory functions (ii) had an age-dependent deleterious effect on spatial memory and visual acuity, and (iii) reduced SOD activity in cortex and hippocampus of old mice. Overall, this preclinical study would seem to confirm some probability of deleterious effect resulting from the central nervous system actions of metformin when presented in a clinically effective dose range and depending on the age at start of intake.

Metformin was without notable effect on food intake, body weight, water intake, and blood glucose levels. These results were not unexpected, as other laboratories have reported that similar metformin doses only slightly reduced food intake and had little effect on body weight [55]. However, at middle age, when mice are at or near their peak weight [57], the metformin treated mice weighed less than their age-matched controls by the end of the study. This finding is in accordance with previous reports of a decrease in body weight within the first month of treatment, together with an increase in lean to fat ratio, in metformin-treated mice fed a high fat diet [39]. This observation is also consistent with increased lean:fat ratio in metformin-treated diabetic patients [58]. While metformin can affect feeding behavior [59, 60], there was no effect of metformin treatment on food intake in our study or others [39], suggesting that the weight loss might be due to increased fat oxidation, a potential new mechanism [61]. Metformin did not change blood glucose at any age in our male mice, which is consistent with other reports in normal, non-diabetic mice [62-65] and in high fat-fed mice [39].

Impairments of reflexes and motor function observed in the control mice were similar to that previously reported [66, 67]. Overall, metformin treatment had no effect on psychomotor function of the mice at any age. Other studies have reported improvements of physical performance with metformin under normal conditions [24] and after high-fat diet intake [39], however the duration of treatment was longer in these studies (5 or 10 months vs. 3 months) which could explain the differences in outcome. Motor performance partially relies on skeletal muscle function, and studies of metformin supplementation in mice have led to contradictory results, with some studies indicating an amelioration of mitochondrial dysfunction [68, 69], and others demonstrating an impairment [70].

Similarly, there was no beneficial effect of metformin supplementation on learning using spatial and non-spatial cognitive tasks (water maze and discriminated avoidance). This finding is consistent with other reports in 2-month-old females from mixed C57BL/6 and 129J background strains [31] and in mice fed a high-fat diet [39], but in conflict with a study in a mouse model of epilepsy that reported an improvement in spatial learning with metformin intake [71]. Additionally, in diabetic subjects, metformin intake increased the risk for cognitive dysfunction when compared to control diabetic patients [33]. While there was no effect on learning, our findings indicated a deleterious effect of the metformin treatment on probe testing, suggesting a specific effect of metformin on retention (memory). While other studies using longer treatment duration and different route of administration have shown improvement of spatial memory [31, 39], another study also reported that metformin treatment worsened memory dysfunction in male mice, but was protective in females [36]. Metformin treatment has been associated with anxiolytic effect [72] which could potentially affect probe performance. However, our data from time spent in the center, an indirect measure of anxiety levels, during spontaneous activity measurements did not show any effect of treatment (all *p*s> 0.413; data not shown).

Visual acuity declined with age and metformin intake furthered the impairment in the old mice. Metformin intake can cause lactic acidosis [73, 74] which has been associated with transient loss of vision or blurred vision [75-77], corrected once the acidosis is managed. Studies suggest that pH level can affect retinal function and could explain the effects of acidosis on visual function [78, 79]. Further studies will be required to identify the molecular mechanisms underlying metformin-associated visual dysfunction in older subjects.

Metformin has been shown to have antioxidant properties via activation of Nrf2 pathway affecting redox status via increased transcription of antioxidant genes [37, 40, 80-82]. Therefore, our study focused on measuring the activity of several enzymes involved in redox homeostasis in response to metformin treatment. Overall, our results showed very little to no effect of metformin treatment on the activity of these enzymes in the liver, hippocampus and cortex. In young mice, the activities of TrxR in the liver and GPx and GST in the hippocampus were increased in response to metformin intake. These findings are in contrast to a study of obese human diabetics where investigators measured GPx activity in plasma before and after treatment [83], and found that metformin treatment decreased GPx activity to that of normal, non-diabetic controls. Our findings would suggest that redox status, though not directly measured using glutathione as a marker, remained unchanged which contrast with a study showing increased reduced glutathione levels in erythrocytes after a 4-weeks metformin treatment in both young and old male rats [40].

In addition, the activity of SOD increased with age in all tissues studied, and was altered by metformin treatment. In the cortex and hippocampus of middle-age and old mice, metformin treatment reduced the activity of SOD. Previously, metformin intake had been associated with an increase in SOD activity in serum from diabetic rats [80]. However, another study showed a decrease in Nrf2, known to activate antioxidant response elements [84], after a 6-month intervention with metformin [39]. Nrf2 augmentation has been implicated in increased longevity and healthspan [85], therefore conversely a decrease in Nrf2 could lead to potentially deleterious outcomes associated with metformin intake. This concept was introduced in a previous study of high dose metformin supplementation which resulted in decreased lifespan in male mice [24], however Nrf2 levels were not measured. Furthermore, a study in neuroblastoma cells indicated that metformin treatment increased production of reactive oxygen species and mitochondrial dysfunction [86]. Based on these studies and ours, the effects of metformin treatment on oxidative burden remain to be elucidated and linked back to functional outcomes in the context of aging.

The results from our study on motor and cognitive function do not support metformin as a potential CR mimetic, as CR has been shown to slow the onset of age-related brain deficits [87, 88]. A recent review postulated that the effects of CR in extending lifespan and ameliorating healthspan by preventing or offsetting the negative consequences of an energy imbalance that would otherwise be observed in individuals consuming more calories [57]. In our study, the metformin-treated mice had similar body weights to the controls, reflecting similar energy balance and could explain a lack of beneficial effect of metformin, if considered a CR mimetic.

Metformin has shown promising value as an anti-aging intervention, however our findings of impaired spatial memory and visual function provide evidence that caution should be applied for the use of this antidiabetic, and that more studies are needed to clearly identify the impact of metformin intake on motor and cognitive functions, with age, sex/gender and dose/duration as primary factors to investigate.
